# How is “stigma” conceived within the biomedical literature on trainee wellness? A directed content analysis

**DOI:** 10.15694/mep.2019.000032.1

**Published:** 2019-02-25

**Authors:** Nicholas Lawson, Adina Kalet

**Affiliations:** 1 Georgetown University Law Center; 2 New York University School of Medicine

**Keywords:** stigma, wellness, wellbeing, discrimination, harassment, disability, burnout, well-being, physician impairment

## Abstract

This article was migrated. The article was marked as recommended.

**Introduction**: Institutional discrimination against trainees with suspected mental disorders has rarely been the focus of medical education research. This study explored whether such neglect might be related to the way in which stigma is conceived by academic medical researchers, and was informed by previous scholarship describing the disability models and stigma agendas believed to predominate within the medical field. The aim was to examine whether researchers might be focusing solely on the ways in which stigma prevents trainees from seeking mental health treatment, while neglecting to address mental health discrimination, as predicted by these scholars’ descriptions.

**Methods**: The authors searched PubMed and Medline for articles using combined terms *wellness* or *wellbeing*; *medical student* or *resident* or *physician;* and *stigma* and *mental*, published between September 12, 2011 and September 12, 2016. Directed content analysis of articles with a primary focus on mental health wellness provided counts of the words *discrimination* and *treatment* and evaluated authors’ recommendations to either reduce discrimination or increase identification and mental health treatment.

**Results**: Of the 25 articles meeting inclusion criteria, 23 used the word *treatment* (median = 7), while only 7 mentioned *discrimination* (median = 0). The authors of at least 20 articles recommended identification and treatment, but none provided any recommendations to reduce institutional discrimination. Three surveys also identified concerns about mental health discrimination that were not taken seriously by the authors of these studies.

**Conclusions**: Current trainee wellness research appears to have largely ignored mental health discrimination as a negative influence. Such findings appear consistent with prior descriptions of a medical model of disability/services agenda to reducing stigma that has been criticized by proponents of a social model/rights agenda. Wellness researchers should take steps to reduce mental health discrimination against trainees and consider limitations of current conceptions of stigma within the medical field.

## Introduction

There is reason to believe that stigma and discrimination against trainees (medical students and residents) with disabilities on the part of medical education institutions is a serious problem. In the US, more trainees filed discrimination lawsuits against medical education institutions on the basis of disability than race, gender, or other category, for both medical students and residents, in the period from 1993 to 2002 ( Minicucci & Lewis, 2003). Yet systematic reviews of international studies on discrimination against trainees in 2007 and 2011 found no studies on discrimination against trainees on the basis of physical or mental disorders and disabilities ( Fnais et al., 2014; Nagata‐Kobayashi et al., 2009). (While terms like *mental illness* or *mental health condition* may be used more commonly in other countries, we use *mental disorder* for the sake of consistency in the rest of this article.)

What might account for this apparent neglect is unclear. But many scholars have suggested that stigma in general is not conceived or problematized within the medical profession in relation to discrimination, but solely as a barrier to engaging in treatment. If so, such conceptions might lead to inadequate acknowledgement of and protection from discrimination for members of the profession with disabilities. In light of these theories and the results from systematic reviews, an empirical evaluation of what stigma means and how the term *stigma* is used within the medical profession seems important and overdue. We now review scholars’ theorized characterizations of how disability and stigma (specifically mental disabilities and mental health stigma) are conceived within the medical field.

### Competing Disability Models and Stigma Agendas

Over the last 50 years ( Haegele & Hodge, 2016), scholars from sociology, law, and other fields have described two prominent models of disabilities such as mental disorders ( Areheart, 2008; Corrigan, 2016; Corrigan & Fong, 2014; Corrigan et al., 2005; Goering, 2010; Shakespare, Lezzoni, & Groce, 2009): the social model and the medical model. The *social model* posits that much of the societal disadvantage associated with mental disorders results not from biological impairments but rather from inaccurate stereotypes, prejudice, and discrimination (see Supplementary File 1 for definitions of these and other relevant terms) against persons with mental disorders. The *medical model*, however, attributes their disadvantage to deficiencies inherent to mental disorders in themselves and to inadequate medical treatment of persons with mental disorders in “fixing” these individuals and making them “normal.”

Proponents of the social model (e.g., advocates and stigmatized individuals) favor what Corrigan ( Corrigan, 2016; Corrigan & Fong, 2014; Corrigan et al., 2005) calls a *rights agenda* to stigma reduction that is similar to that pursued by racial, ethnic, sexual, and other minorities that have been discriminated against and denied civil rights. But proponents of the medical model (e.g., healthcare providers) generally support a *services agenda* to reducing stigma that aims to identify and treat individuals suspected of having mental disorders and generally views stigma as problematic insofar as it prevents persons with mental disorders from seeking or engaging in mental health treatment (see Table 1 for a comparison of the models and agendas).

**Table 1.** Two agendas for reducing the stigma associated with mental disorders *

Agenda	Rights Agenda	Services Agenda
Proponents	• Victims of stigmatization and discrimination; advocates demanding social justice	• Medical and mental health service providers and their associated organizations
Rationale	• Due to stigma, people are not able to achieve important personal goals related, for example, to work, independent living, and health	• Due to stigma, people do not seek out or remain in treatment
Task	• Decrease stigma so people are better able to avail opportunities related to work, independent living, and health	• Decrease stigma in order to increase care seeking and engagement
Assumptions	• Stigma is a social justice problem • Persons with mental disorders and other stigmatized individuals have generally not earned the negative stereotypes to which they are assigned • Mental health services may be helpful but will not address the underlying causes of stigmatization • Stigma persists in spite of treatment	• Stigma is a public health problem • There is a kernel of truth to the negative stereotypes that are associated with persons with mental disorders • Service providers can fix persons with mental disorders and make them “normal” • Treatment will reduce stigma
Associated disability models	• Social	• Medical
Highlights contributions of which groups to stigma	• Institutions and individuals in positions of authority and power	• Stigmatized groups and individuals

* Adapted from Areheart, 2008; Corrigan, 2016; Corrigan & Fong, 2014; Corrigan et al., 2005; Goering, 2010; Haegele & Hodge, 2016; Shakespare, Lezzoni, & Groce, 2009.

Critics of the latter model/agenda have raised the concern that it may actually exacerbate stigma ( Areheart, 2008; Corrigan, 2016; Corrigan & Fong, 2014; Corrigan et al., 2005; Goering, 2010). Overemphasizing the deficiencies or dangers assumed to accompany mental disorders in order to increase treatment seeking may promote additional prejudice ( Areheart, 2008; Corrigan, 2016; Corrigan & Fong, 2014; Corrigan et al., 2005; Haegele & Hodge, 2016). A related concern is that focusing too much on stigma as a barrier to treatment may lead to neglect of discrimination against persons with suspected mental disorders. Neglecting to address discrimination may lead to the exclusion of otherwise qualified persons with mental disorders within the profession and society, as well as to adverse mental health ( Link & Hatzenbuehler, 2016) for those who experience discrimination.

### “Stigma” in the Wellness Literature

These issues take on additional significance in the context of current debates about wellness programs and initiatives, which are strongly opposed by many disability rights advocates but strongly supported by occupational health organizations and medical leaders ( Anderson et al., 2016; Bagenstos, 2017; Kirkland, 2014; Madison, 2016). These advocates allege that wellness initiatives may result in further stigmatization and discrimination against persons with mental disorders and disabilities. However, there has so far been inadequate attention and research on these possible drawbacks ( Madison, 2016). Medical leaders and wellness researchers also frequently cite stigma as an important negative influence on wellness but seem to problematize stigma very differently.

Wellness has also become a hot topic in medical education research in part because of concerns that overworking trainees may adversely affect their educational experience, their health, and patient safety outcomes. Much of the medical literature on wellness concerns trainee or physician wellness. *Wellness* is a term used interchangeably with *wellbeing*, and has been variously defined, but it loosely refers to health, or quality of work and personal life (Brady et al., 2017). It is currently unknown how medical researchers on trainee wellness problematize stigma, whether their stigma conceptions encompass discrimination, and whether they see discrimination as relevant to wellness for trainees or for ethical and legal reasons.

### Study Purpose

This study sought to better characterize how stigma is conceived within the medical literature on trainee wellness. The study was designed to answer the following research question: do authors of articles on mental health wellness of trainees problematize mental health stigma exclusively as a barrier to their engaging in mental health treatment (consistent with a services agenda), while neglecting to address discrimination against trainees with suspected mental disorders (consistent with a rights agenda)? We hoped to answer this question through word counts of *discrimination* and *treatment* and detailed evaluations of authors’ recommendations. We hypothesized that these studies would largely focus on stigma in relation to mental health services and treatment rather than discrimination and rights, as predicted from the relevant literature.

## Methods

We adhere to the standards for reporting qualitative research in O’Brien et al. (2014), its supplements, and associated references.

### Research Paradigm and Qualitative Approach

We designed this study from a post-positivist point of view, which seeks to establish probable truth by testing hypotheses and using well-defined variables with precise implementation, in order to limit bias of results ( Bunniss & Kelly, 2010). Our purpose was aggregative (testing predefined concepts) rather than configurative (generating theory).

In contrast to positivist paradigms, which posit that knowledge can be neutral or value-free, post-positivist paradigms acknowledge that our attempts to understand the world are inherently susceptible to bias, and aim only to generate supporting or non-supporting evidence for a theory. This is seen in our intentionally limited search strategy. We did not attempt to identify all articles primarily related to mental health wellness of trainees. Instead, we hoped to identify enough to generate support (or lack of support) for our hypothesis that this literature has *probably* been focusing on how stigma can prevent seeking treatment (consistent with a medical model of disability/services agenda to reducing stigma), while neglecting to address institutional discrimination (consistent with a social model of disability/rights agenda to stigma reduction).

We selected directed (deductive) content analysis, a thematic analysis that is one type of qualitative evidence synthesis, as our qualitative approach. Directed content analysis proceeds from an *a priori* framework, and uses coding and categorizations, with direct implications for research and policy ( Hsieh & Shannon, 2005). While other types of content analysis and qualitative research often occur as an iterative process (Brady et al., 2017), in directed content analysis, the coding strategy is derived from theory and defined before data analysis ( Hsieh & Shannon, 2005). We chose this approach in part because our purpose was testing a hypothesis/theory (based on the models/agendas previously described), and evaluated articles’ relative consistency with one of two discrete categories or models/agendas. We also expected that our research might be viewed skeptically by those not familiar with these models/agendas, and this approach is conceptually simple and can provide transparent and confirmable results. Thus, our decision pre-study was to code authors’ recommendations into one of two discrete categories based on preexisting theory (one based on a social model/rights agenda, and the other based on a medical model/services agenda), for example, reflects an approach with this method.

### Researcher Characteristics and Reflexivity

The coding and analyses were performed by the authors, both of whom had experience in qualitative methods and had previously performed directed content analyses. The first author (NL) has experience in research related to mental health stigma within the medical profession as a psychiatry resident, and is a current law student. The second author (AK) is co-director of a medical education research unit at an academic medical center, has tenure, and is a general internist with an MPH with previous research and publications on trainee wellness. They have collaborated on prior publications related to mental health discrimination and relevant law in medical education. Neither author has ever functioned as teacher or supervisor or held any position of authority over the other. Their combined publications reflect a range of perspectives on issues potentially relevant to this topic.

### Sampling Strategy

The sampling strategy was developed by both authors and finalized in September 2016. We selected academic medical journal articles on wellness as the focus of our research in part because they represent a relatively discrete body of written empirical data that is publicly available. Organizational or government websites and documents have been the focus of previous research by the authors, and we felt their inclusion here would dilute the impact of our findings for medical researchers. Our decision pre-study to evaluate publications of current academic medical researchers also seemed more likely to emphasize relatively more powerful members of the profession (e.g., medical leaders) than those typically problematized as sources of mental health stigma originating from the profession (e.g., trainees, nurses, social workers, and admission clerks [ Henderson et al., 2014]), and many stigma theorists and rights advocates believe that stigma originates from those in relative positions of power (e.g., Link & Phelan, 2014).

We limited our search to articles published between September 12, 2011, and September 12, 2016, to capture the stigma conceptions of those likely to be active and influential within the medical field and likely to be able to shape the current stigma approach to mental health wellness. Confining our search to a smaller number of documents also allowed us to analyze the articles selected with greater intensity and present our results to readers in greater detail.

We searched PubMed and Medline using all fields (not MeSH terms) to identify potentially relevant journal articles with the combined words *wellness* or *wellbeing*; *medical student* or *resident* or *physician;* and *stigma* and *mental.* For a number of reasons, we limited our search to these specific words and did not attempt to identify all articles potentially relevant to wellness. First, *wellness* is not yet well-defined (Brady et al., 2017), and we wished to avoid making subjective assessments of which articles did or did not primarily concern wellness as much as possible. Second, the primary focus of this study was on problematic stigma conceptions and ramifications rather than wellness per se. And we also believed the search words chosen here could still identify a sizeable number of articles on wellness that would allow for meaningful inferences related to the targeted literature and would allow us to test and help answer the research question.

**Figure 1.**
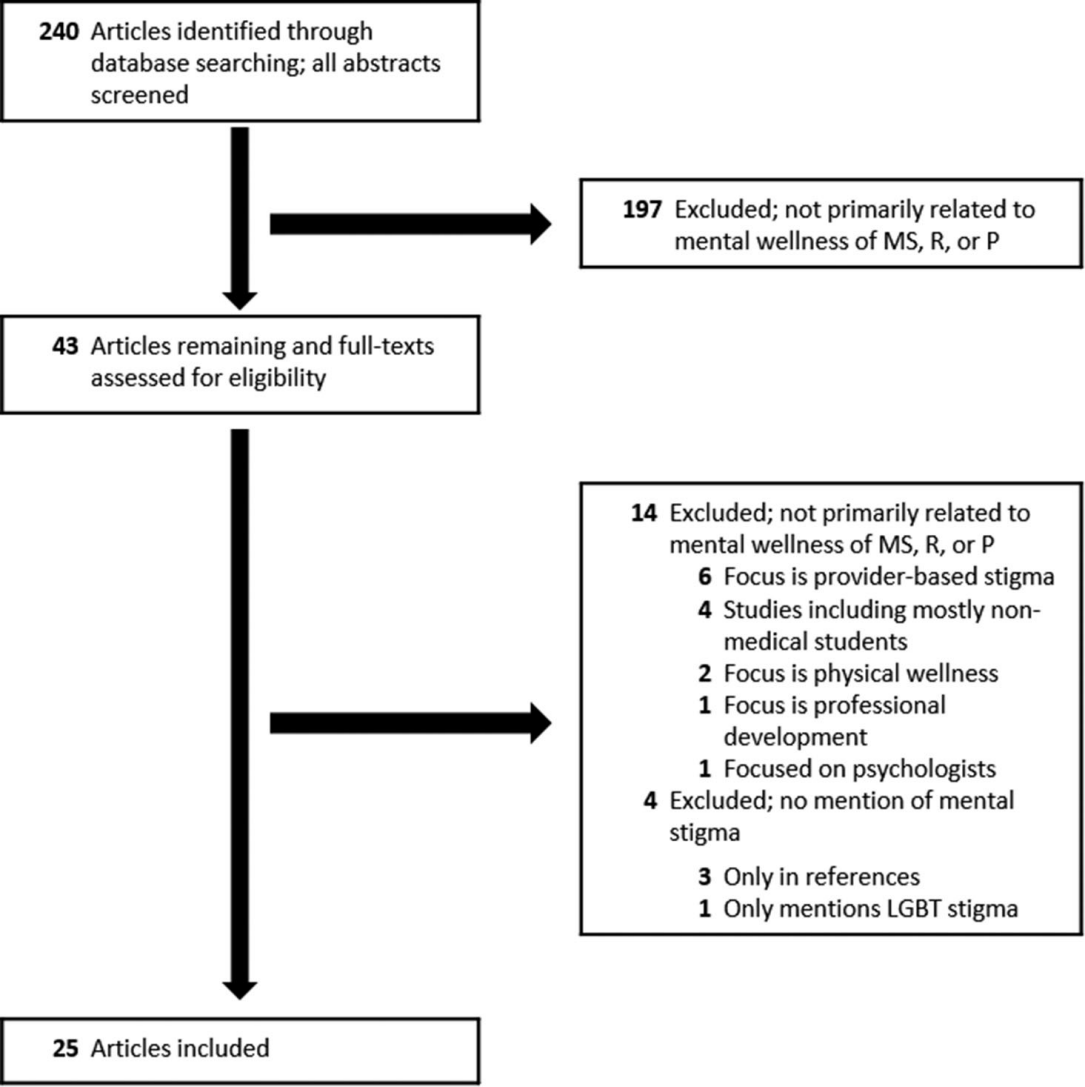
Search strategy used to select journal articles primarily concerning medical student, resident, or attending physician mental wellness that referred to mental health stigma, and published between September 12, 2011, and September 12, 2016.

Abbreviations: MS indicates medical students; R, residents; P, attending physicians; LGBT, lesbian, gay, bisexual, or transgender

We included attending physicians because wellness studies often include respondents or recommendations that apply to both trainees and attending physicians. We also confined the search to journal articles, excluding abstracts and comments in response to other articles, that were written in English. Within these parameters, we erred on the side of inclusion at each stage of the search strategy (see Figure 1) so as to limit bias and included studies regardless of type.

We screened all abstracts (and full-texts for those documents with no abstracts available) and excluded those articles which could reasonably be determined were not primarily related to the mental wellbeing of trainees or attending physicians (see Figure 1). Full-text reviews then eliminated those articles which were not relevant or which did not mention stigma* in the body of their text (see Supplementary File 2 for a list of all 240 articles resulting from the initial database search, inclusion status at each phase of screening, and explanations).

### Data Analysis

Word counts of stigma* provided a rough estimate of its relevance to the article overall. To determine whether stigma was problematized as a barrier to treatment (consistent with a medical model of disability/services agenda to stigma reduction), we searched the article texts for any references to a particular type of stigma concerning *treatment carryover*, or “belief that public knowledge that an individual has received medical or psychological treatment for a stigmatized condition and/or status reduces the status of that individual in the larger community” ( Pescosolido & Martin, 2015:p.97) (see Supplementary File 3 for text mentioning stigma related to treatment carryover).

We also performed word counts of the terms *discrimination* and *treatment* to provide a rough but empirical index of the articles’ focus. Higher counts of *discrimination* would be consistent with a social model of disability/rights agenda to stigma reduction, while higher counts of *treatment* would be consistent with a medical model of disability/services agenda to stigma on the part of these authors.

Our analyses of authors’ recommendations was more complex. Both authors independently reviewed the articles using a structured approach that considered two main factors when deciding whether a statement reflected a recommendation: position in the text and the presence of evaluative language. We initially confined our analysis of recommendations to statements made in subsections identified as conclusions, either at the end of the paper or in the abstract; discussion; implications; comments, or learning points. When either an article had no such subheadings or our analysis of these sections did not identify one of the two recommendation types, we considered general placement in the final paragraph of the article or the use of evaluative words (e.g., *should*, *need*). The presence of these words was necessary, but not sufficient, in order to code as a recommendation.

Our classification of recommendations into types, however, did not depend on the presence of any particular language. In our attempts to identify recommendations to reduce institutional discrimination (consistent with a social model/rights agenda), for example, we looked for a range of suggestions such as changing discriminatory laws or policies (e.g., related to physician impairment) or enforcement of existing policy related to discrimination; training faculty and trainees who might sit on institutional grievance committees about antidiscrimination laws; collecting and publicly disseminating data regarding trainees’ allegations of discrimination in order to facilitate comparisons between programs; or any other suggestions that could potentially be construed as a recommendation to reduce institutional discrimination.

Our process for classifying recommendations into types was as follows: both authors independently reviewed the subsections of text described previously for recommendations, while NL also transcribed the text in each article most likely to reflect each type of recommendation into a summary document (see Supplementary File 4 for text excerpts potentially reflecting author recommendations). The authors then discussed their impressions, reviewed candidate statements, and documented their rationales for all coding decisions in full. This approach to coding, while structured, afforded sufficient flexibility to consider the articles’ statements in appropriate context, given the complexities involved in this analysis. We completed all coding and analysis by June 2017.

### Techniques to Enhance Trustworthiness

We developed detailed procedures to analyze journal articles using specific words, in order to limit the influence of authors’ biases on the results. We achieved triangulation in a number of ways. We designed our study from a theoretical framework (based on the models/agendas described previously) informed by sociology, law, disability studies, and other disciplines. We also searched for any evidence that might not be consistent with our hypothesis. We reviewed all articles independently and discussed our impressions from different perspectives and experience. We have presented detailed information on our coding and rationale to allow readers to evaluate our results on their own in the Supplementary Files.

## Results/Analysis

From our initial database search, we identified 240 potentially relevant journal articles (see Figure 1), of which 25 met the inclusion criteria and were included in the study (see Supplementary File 5 for characteristics of the included articles and results). Countries represented by more than one article were the US (15 articles or 60% of the total), the UK (4; 16%), Spain (2; 8%), and Australia (2; 8%). The most commonly represented journals were *Academic Medicine*, with five articles, followed by *Academic Psychiatry*, with three, and *BMJ Case Reports*, with two. The majority, or 60% (15/25), were cross-sectional surveys, and three surveyed medical students for their concerns about mental health discrimination in the medical profession. Most of the articles, or 72% (18/25), concerned medical students; 28% (7/25) concerned residents; and 40% (10/25) pertained to attending physicians (some articles focused on more than one group).

Word counts for *stigma* per article ranged from 1 to 88 (mean = 20; median = 8), and all 25 articles used the term *stigma* when describing a barrier to mental health treatment. Almost all or 92% (23/25) of the articles mentioned *treatment* (consistent with a medical model/services agenda), and the word count sum for all 25 articles was 269 (mean = 11; median = 7). In contrast, only 28% (7/25) mentioned *discrimination* (consistent with a social model/rights agenda), which had a word count sum of 34 (mean = 1; median = 0).

Our analyses of author recommendations found at least 20 (80%) recommending identification and treatment (consistent with a medical model/services agenda). One additional paper ( Estabrook & Christianson, 2013) implied such a recommendation but did not use sufficiently clear and specific language to be classified as such. Remarkably, we found no articles with author recommendations to reduce institutional discrimination (the presence of which would be consistent with a social model/rights agenda). We review this result by reporting on the seven articles which included the word *discrimination* at some point in their texts. Though our analysis of recommendation types was not limited to the use of this word, it is hoped that reviewing the following text excerpts will support the validity of our coding and our specific findings that no authors made any recommendations to reduce institutional discrimination.

### Articles Mentioning Discrimination

One sentence came close to a recommendation for decreasing institutional discrimination, and it was repeated verbatim in the conclusions of two articles from Hankir et al. (2014a, 2014b).

Antistigma work targeting specific groups, such as healthcare staff, or strategies that empower individuals facing discrimination, are likely to play a key role in reducing the impact of stigma ( Hankir et al., 2014a:p.S95).

The statement and its context, however, did not indicate whether the authors referred to discrimination faced by persons with mental disorders in general or by members of the medical profession. They also did not provide any examples or guidance about what kind of strategy they believed would empower individuals facing discrimination.

Only one other article ( Wimsatt, Schwenk, & Sen, 2015) used the word discrimination when making a recommendation. The authors, however, left unclear whether they posit actual experiences of discrimination or only *beliefs* about discrimination.

Educational experiences that target specific perceptions, experiences, and beliefs about personal weakness, discrimination, and devaluation might prove useful in addressing medical student beliefs about depression and its treatment as part of a professionalism curriculum ( Wimsatt, Schwenk, & Sen, 2015:p.711).

The problem, according to these and similar statements, is not actual discrimination on the part of institutions, but rather with medical students’ beliefs about discrimination, and these beliefs are relevant primarily as part of their training in professionalism.

Only one article ( Dyrbye et al., 2015a) speculated that institutional discrimination against members of the medical profession might be more than just an unfounded fear:

This finding suggests that confidentiality breaches and discrimination may actually occur, rather than solely being feared as a consequence for students with burnout who seek help ( Dyrbye et al., 2015a:p.965).

This possibility, however, was not further addressed by these authors. Additional context from the article suggests that the authors’ speculation might refer to possible discrimination against medical students by other medical students or their immediate supervisors, but may not have extended to directors of medical student education, deans, residency program directors, or other more influential and powerful institutional players.

Three other articles used the word *discrimination* only once, in their introductions. Of these, only Martinez et al. (2016) placed the word specifically in the context of the medical profession:

Barri-ers to care for health professionals may include lack of time [and] fear of stigmatization and discrimination ( Martinez et al., 2016:p.91).

The statement similarly problematizes beliefs about discrimination, rather than discrimination itself, and presents those beliefs as significant insofar as they prevent seeking treatment.

### Articles Citing Medical Student Concerns

Three of the 15 cross-sectional surveys ( Cheng et al., 2013; Dyrbye et al., 2015a; Wimsatt, Schwenk, & Sen, 2015) asked medical students for their views regarding the presence of discrimination against trainees with real or suspected mental disorders.

Cheng et al. (2013) asked 1,010 medical students five questions related to so-called *personal stigma*, two of which explicitly asked for their views regarding employment discrimination, and whether they thought they might be discriminated against by employers (i.e., program directors in their case) if they experienced depression, finding that 33.6% believed their employers would “dismiss me before others,” and 37.8% “would pass over my application.” The authors did not make any recommendations to reduce institutional discrimination. Instead, they focused on comparing responses between students reporting a history of depression and those who did not; they found no statistically significant differences for any personal stigma variables between these groups. At the end of their discussion, the authors concluded only with the need for improved support and treatment.

High levels of personal stigma may mask the clinical presentation of a mental illness and cause delays in seeking treatment. The identification of groups more susceptible to depression and stigmatizing views, such as those displaying high levels of stress warrants greater consideration for tailored support services to promote better views towards depression support amongst medical students ( Cheng et al., 2013:p.689).

Dyrbye et al. (2015a) surveyed 873 medical students and found that 50.3% believed that residency program directors would pass over their application if they were believed to have a mental health problem such as depression or anxiety, and that 46.4% believed that faculty, residents, or deans would see them less favorably. The authors made no suggestions to address the implied discrimination by program directors, faculty, residents, or deans. Instead, they pointed to differences between the responses of medical students.

Our finding of increased stigma scores among distressed students (suffering from depression and burnout) points to distorted perceptions that work against the distressed individual’s ability to frame the problem and optimally problem solve ( Dyrbye et al., 2015a:p.965).

Wimsatt, Schwenk, and Sen (2015) surveyed 769 medical students and found that 53.3% believed that most people think depressed medical students will provide inferior treatment to patients; 50.3% believed their residency applications would become less competitive, and 85.9% believed it would be risky to reveal being depressed on an application. The authors similarly did not consider residency programs as the problem, but responded to these results with the following recommendations:

Our results identify depression stigmatization as perceived by medical students. Although stigmatization may raise concern that public disclosure of a medical student’s depression could compromise a student’s status, such concern should not outweigh the importance of confidential and robust care for depressed students ( Wimsatt, Schwenk, & Sen, 2015:p.711).

## Discussion

This study provides important insights into the way mental health stigma is problematized within the literature on trainee wellness. Our analysis of articles meeting inclusion criteria found that the overwhelming majority recommended treatment (consistent with a medical model of disability/services agenda to reducing stigma), but none that recommended reduction of institutional discrimination (whose presence would be consistent with a social model of disability/rights agenda to reducing stigma). Only seven articles actually used the word *discrimination.* Text excerpts from all three articles that reported significant concerns about mental health discrimination from medical students ( Cheng et al., 2013; Dyrbye et al., 2015a; Wimsatt, Schwenk, & Sen, 2015) demonstrate that trainee concerns were not taken seriously by these authors. Two even suggested that the trainees’ concerns were either a sign of mental illness ( Cheng et al., 2013) or else reflected “distorted perceptions” attributable to their own underlying psychopathology ( Dyrbye et al., 2015a) and not accurate perceptions or experiences, further proving the point of these authors about the need for more identification and treatment interventions. Attributing discrimination concerns to trainees’ “irrational” fears and stigma views may have allowed these authors to avoid acknowledging the possibility that these deans and residency directors might be discriminating against them.

Our findings suggest that these researchers have problematized stigma as a barrier to treatment, consistent with a medical model of disability/services agenda to reducing stigma. Our results indicate that in this literature, stigma is not problematized as a social injustice that results in discrimination. That the few articles that included the word *discrimination* problematized *beliefs* related to institutional discrimination instead of discrimination itself is also consistent with this characterization. That said, our study was not explicitly designed to evaluate these articles’ consistency with features of the models or agendas described in Table 1; additional research to fully evaluate the applicability of these descriptions to the medical field more broadly could be informative.

### Implications

Disability discrimination is a real problem at medical education institutions, as illustrated by the prevalence of disability discrimination lawsuits by medical trainees ( Minicucci & Lewis, 2003). Failing to acknowledge disability discrimination and its implications, along with viewing mental health stigma solely in relation to treatment within the medical profession, may contribute to a number of undesirable outcomes. First, attributing *stigma* as a problem of irrational beliefs on the part of trainees and attending physicians assumed to be “in denial” or “lacking insight” may facilitate coerced mental health treatment ( Lawson & Boyd, 2018a, 2018b) of persons who may not benefit from treatment referrals and may be harmed. In addition, denial of possible discrimination and inappropriate referrals for mental health treatment may have a censoring effect on trainees and attending physicians voicing concerns or blowing the whistle on institutions ( Lawson & Boyd, 2018a, 2018b). Such referrals may contribute to unjust cultures and unsafe conditions for patient care.

Current medical conceptualizations of *stigma* may lead to neglect of institutional discrimination and pose a particular problem in the context of wellness (wellbeing) programs and initiatives within medicine and many other educational or occupational settings. Kirkland (2014), a disability rights advocate, writes:

The increased focus in law, business, and culture on personal wellness overwhelms the political conversations about health and health care provisioning so that designing structural or redistributive solutions is no longer feasible because the notion that individual behavioral changes can fix everything will be so hegemonic ( Kirkland, 2014:p.976).

Kirkland also observes the potential for such “focus” to negatively impact many persons other than those with real or suspected disabilities, noting the “power of wellness to create and reproduce hierarchy, to promote homogeneity, narrow-mindedness, and moralism about how to live one’s life, and to cover for discrimination based on health, weight, income, age, pregnancy, and disability” ( Kirkland, 2014:p.971).

Medical leaders have argued that these and other similar critiques are not relevant to current efforts to improve trainee and attending physician wellness ( Meeks, Jain, & Herzer, 2019; Tawfik et al., 2018). Yet many disability rights criticisms of wellness (e.g., its potential to “institutionalize disability bias” [ Basas, 2014;p.1035]) are ideological and not limited to a particular setting. These critiques take issue with institutional efforts to coerce participation in treatments while dismissing the concerns of those unwilling to participate and ignoring discrimination.

Medical leaders also have argued that they are committed to structural interventions and often note recent systematic reviews of wellness interventions for trainees and attending physicians finding that organizational approaches (e.g., workload changes) are more effective than those focused on individuals (e.g., identifying and treating those with suspected mental disorders) ( Panagioti et al., 2017; West et al., 2016), which often seem to blame individual employees for the problem ( Panagioti et al., 2017). Yet most (e.g., Thomas, Ripp, & West, 2018) propose largely individually-based interventions; do not consider potential unintended consequences; tout statistics regarding the efficacy of their approaches that strain credulity ( Weeks, 2018); misleadingly claim that burnout or poor wellbeing are meaningful causes of medical errors or poor patient care ( Lawson, 2018; Tyssen, 2018); and do not address disability discrimination.

Many policies that discriminate against trainees or attending physicians with disabilities remain ( Dhrewsbury, Mogensen, & Hu, 2018; Lawson & Boyd, 2018a, 2018b). There are even some expansions-e.g., the Accreditation Council for Graduate Medical Education’s (ACGME) Common Program Requirements on resident wellbeing (defining wellbeing as a component of competence and listing depression and substance abuse as indicative of poor wellbeing), which have just been implemented ( ACGME, 2017). This is not acceptable.

Addressing mental health discrimination against trainees and attending physicians might improve their mental health. It might also improve patient safety, quality of care, and productivity by creating just cultures. But perhaps the most important reason to reduce discriminatory policies and practices in medical education institutions is that trainees are human beings with human rights, and because including persons with disclosed mental disorders within the medical field might break a glass ceiling that could empower many individuals with mental disorders.

### Limitations

One limitation to this study is its relatively narrow focus on wellness research published within the last five years. This reflected a deliberate choice on our part to evaluate the current approach to research and stigma among present-day leaders who are likely to be continuing active roles with influence within the medical field. In addition, these specifications left us better able to rigorously evaluate a discrete body of the literature in order to ensure confirmability and present our findings in greater complexity. Our search strategy and specific search words were not intended to capture every article of possible relevance to wellness, which has only loosely been defined and was not central to our research interest. However, we believe our findings provide meaningful information about this literature that permits a good test of our hypothesis and illuminates how stigma is being conceptualized in this research. Our results may not necessarily generalize to the medical literature overall. But the results of systematic reviews of a sizeable portion of the relevant literature (e.g., Fnais et al., 2014; Nagata‐Kobayashi et al., 2009)-finding no studies on mental health discrimination against trainees despite many allegations and lawsuits-suggest that a similar pattern may exist in the overall medical literature.

Our initial analysis generally consisted of only a few coding decisions for each of the 25 articles meeting the inclusion criteria. Our use of word counts, while simple, provided an empirical measure and important information in addition to our comprehensive assessments of author recommendations. We also decided not to code for the presence of various stigma types, such as described by Pescosolido and Martin (2015), because they are generally not known within the medical field; further investigation of such stigma types might provide additional perspective.

While analytic bias in directed content analysis is an acknowledged limitation of the approach, we took a number of steps (see Techniques to Enhance Trustworthiness in Methods) to limit bias; extensive documentation of our coding rationale is available as Supplementary Files. Given our methods, our results are clear, and it seems unlikely that any alterations in the analytic approach would have substantially changed the overall findings.

There is no question that mental health stigma that prevents seeking help when needed can be a serious problem. But an overemphasis on mental health stigma as a barrier to treatment (consistent with a medical model/services agenda to stigma reduction) to the exclusion of addressing discrimination and promoting basic human rights for physician-trainees (which would be consistent with a social model/rights agenda to stigma reduction) is also a serious problem.

## Conclusion

Results of our analyses of published research on trainee wellness suggest that the authors of these studies have problematized mental health stigma primarily as a barrier to treatment, while neglecting to consider its discriminatory impact and, in fact, failing to acknowledge actual discrimination, despite the high prevalence of discrimination lawsuits against medical education institutions. The unbalance of the articles overall and the problematic interpretations of medical students’ feedback displayed in some of these articles suggest the need for critical reflection among editors and reviewers as well as the medical hierarchy about what may have led to these results and why the responses from student surveys were so easily dismissed. While our findings indicate the need for a change in approach, they may also provide a potential solution to the problem of stigma, wellness, and discrimination within medicine. Institutional leaders may only need to do no harm, to change their own policies, and avoid discrimination in order to improve the training environment.

## Take Home Messages

• Authors of trainee wellness articles currently appear to view stigma as problematic insofar as it prevents engaging in treatment, but not as a social injustice that results in discrimination.
• Authors did not acknowledge discrimination and so made no recommendations to reduce institutional discrimination against trainees with suspected mental disorders and disabilities.
• Results were consistent with previous descriptions of the overwhelming predominance of a medical model of disability/services agenda to reducing stigma.

## Notes On Contributors

Nicholas D. Lawson, MD, is a former psychiatry resident and current full-time law student at Georgetown University Law Center in Washington, DC, USA. https://orcid.org/0000-0003-3333-0922

Adina L. Kalet, MD, MPH, is Arnold P. Gold Professor of Humanism and Professionalism, Co-Director of Program on Education, Innovations, and Research (PrMEIR), Division of General Internal Medicine and Clinical Innovation, New York University School of Medicine, New York, NY, USA. https://orcid.org/0000-0003-4855-0223
