# Supplementary material for: How is “stigma” conceived within the biomedical literature on trainee wellness? A directed content analysis
Source: MedEdPublish (2016). 2019 Feb 25;8:32. [Version 1] doi: 10.15694/mep.2019.000032.1 (PMC10712616; doi:10.15694/mep.2019.000032.1)
Supplement: Peer review history

# Reviewer report: Reviewer response for version 1

**DOI:** 10.21956/mep.19739.r29590

**Published:** 2019-06-21

**Authors:** Hassaan Waqar

**Affiliations:** 1 St Helens and Knowsley Teaching Hospitals NHS Trust – Lead Employer

This review has been migrated. The reviewer awarded 3 stars out of 5 An important area of research is explored by the authors regarding stigma and how it is conceived within the literature. The literature review identifies 25 articles which are selected for analysis and discussed in this article. The article identifies important areas of further research and highlights factors which need to be addressed in order to reduce the effect of the stigma. An area in which the article could have been improved is by discussing in more detail methods which could be employed in order to reduce the effect of stigma.

Reviewer Expertise:

NA

No decision status is available

# Reviewer report: Reviewer response for version 1

**DOI:** 10.21956/mep.19739.r29589

**Published:** 2019-03-13

**Authors:** Judy McKimm

**Affiliations:** 1 Swansea University

This review has been migrated. The reviewer awarded 4 stars out of 5 This is an interesting review based on the two competing models of how disability/mental health/wellness is viewed by those in medicine and medical education. Unsurprisingly, the review finds that the biomedical model prevails and that a rights based perspective is much less prominent. It leads us on to consider how best to support people with disability and mental health disorders (maybe we shouldn't call them disorders!!) and the subsequent stigma that occurs because of this. It raises some interesting questions around 'wellbeing' and how we should frame support to promote people's rights rather than using a deficit model. As the authors say, more research is needed here. An interesting paper.

Reviewer Expertise:

NA

No decision status is available

# Reviewer report: Reviewer response for version 1

**DOI:** 10.21956/mep.19739.r29588

**Published:** 2019-03-04

**Authors:** Peter Dieter

**Affiliations:** 1 AMSE

This review has been migrated. The reviewer awarded 4 stars out of 5 The paper describes how „stigma“is conceived within the biomedical literature on trainee wellness. The results are based on a literature research.The results show that trainee wellness research largely ignores mental health discrimination as a negative influence.The authors recommend that there might be a need for editors and reviewers for a more critical reflection,and that institutional leaders should change their policies and avoid discrimination to improve the training environment

Reviewer Expertise:

NA

No decision status is available

# Reviewer report: Reviewer response for version 1

**DOI:** 10.21956/mep.19739.r29587

**Published:** 2019-02-28

**Authors:** Susan Haney

**Affiliations:** 1 Curry General Hospital

This review has been migrated. The reviewer awarded 5 stars out of 5 Important, timely, and thought-provoking review of the medical literature, discussing the personal and societal costs of stigma-perpetuating agendas (such as those currently promoted by medical licensing boards and physician health programs in their disciplinary/exclusionary practices towards physicians they believe have a history of mental health diagnosis and/or treatment). These costs include both barriers to accessing care and subsequent discriminatory impact of receiving (or having received) care for these common but treatable illnesses.

Reviewer Expertise:

NA

No decision status is available
